# Supplementary material for: Modelling Skylarks (Alauda arvensis) to Predict Impacts of Changes in Land Management and Policy: Development and Testing of an Agent-Based Model
Source: PLoS One. 2013 Jun 6;8(6):e65803. doi: 10.1371/journal.pone.0065803 (PMC3675089; doi:10.1371/journal.pone.0065803)
Supplement: Supporting Information S4 — The skylark ODdox as a zipped archive. (ZIP) [file pone.0065803.s004.zip › Skylark_ODdox/functions_0x67.html]

ALMaSS Skylark ODdox: Class Members


ALMaSS Skylark ODdox
 2.0


- Main Page
- Related Pages
- Classes
- Files

- Class List
- Class Index
- Class Hierarchy
- Class Members

- All
- Functions
- Variables
- Typedefs

- a
- b
- c
- d
- e
- f
- g
- h
- i
- j
- k
- l
- m
- n
- o
- p
- r
- s
- t
- u
- v
- w
- x
- y
- z
- ~

Here is a list of all class members with links to the classes they belong to:

### - g -

- Garden()
  : Garden
- GeneticsResultsOutput()
  : Population\_Manager
- Get()
  : RasterMap
- GetArea()
  : LE
  , Farm
- GetBadWeather()
  : Skylark\_Adult
- GetBaitStartDate()
  : RodenticideManager
- GetBLtype()
  : BaitLocation
- GetBorder()
  : LE
- GetBugPercentA()
  : CropData
- GetBugPercentB()
  : CropData
- GetBugPercentC()
  : CropData
- GetBugPercentD()
  : CropData
- GetCanGrow()
  : EdgeGrowingPoints
- GetCattleGrazing()
  : LE
- GetCentroidX()
  : LE
- GetCentroidY()
  : LE
- GetCountryDesignation()
  : LE
- GetDayDegrees()
  : LE
- GetDayInMonth()
  : Calendar
- GetDDDegs()
  : Weather
- GetDeadBiomass()
  : LE
  , VegElement
- GetDigestability()
  : LE
  , VegElement
- GetElementType()
  : LE
- GetFarmIndex()
  : FarmManager
- GetFarmNumber()
  : Farm
- GetFileType()
  : LE
- GetFirstCrop()
  : CropRotation
- GetFirstCropIndex()
  : Farm
- GetFirstDate()
  : Crop
  , Farm
- GetFirstYear()
  : Calendar
- GetFledgelingEM()
  : Skylark\_PreFledgeling
- GetFood()
  : Skylark\_PreFledgeling
  , Skylark\_Female
  , Skylark\_Male
- GetGlobalRadiation()
  : Weather
- GetGreenBiomass()
  : LE
  , VegElement
- GetHeightDiff()
  : CropData
- GetHigh()
  : LE
- GetHour()
  : Calendar
- GetID()
  : RasterMap
- GetInsectPop()
  : LE
  , VegElement
- GetIntensity()
  : Farm
- getkey()
  : CfgBase
- GetLAGreen()
  : LE
  , VegElement
- GetLAgreenDiff()
  : CropData
- GetLastTreatment()
  : LE
- GetLastYear()
  : Calendar
- GetLATotal()
  : LE
  , VegElement
- GetLAtotalDiff()
  : CropData
- getlevel()
  : CfgBase
- GetMagicP()
  : RasterMap
- GetMap()
  : RodenticideManager
- GetMapDensity()
  : PositionMap
  , SimplePositionMap
  , ScalablePositionMap
- GetMapDensity32()
  : PositionMap
- GetMapDensity5x5()
  : PositionMap
- GetMapIndex()
  : LE
- GetMapPositive()
  : SimplePositionMap
  , ScalablePositionMap
  , PositionMap
- GetMapPositiveB()
  : PositionMap
- GetMapTwin()
  : RodenticideManager
- GetMapValid()
  : LE
- GetMapValue()
  : MovementMap
  , MovementMap16
  , IDMap
  , SimplePositionMap
  , ScalablePositionMap
  , PositionMap
- GetMass()
  : BaitLocation
- GetMaxX()
  : LE
- GetMaxY()
  : LE
- GetMConstants()
  : LE
- GetMDates()
  : LE
- GetMeanTemp()
  : Weather
- GetMgtLoopDetectCount()
  : LE
- GetMgtLoopDetectDate()
  : LE
- GetMigrationMortality()
  : Skylark\_Female
  , Skylark\_Male
- GetMinute()
  : Calendar
- GetMinX()
  : LE
- GetMinY()
  : LE
- GetMonth()
  : Calendar
- GetNextCrop()
  : CropRotation
- GetNextCropIndex()
  : Farm
- GetNextCropStartDate()
  : Farm
- GetNextSeed()
  : Landscape
- GetNumCrops()
  : CropData
- GetNutStatus()
  : CropData
- GetNutStatusExt()
  : CropData
- GetOldDays()
  : LE
- GetOwner()
  : LE
- GetOwnerFile()
  : LE
- GetOwnerIndex()
  : LE
- GetPesticideCell()
  : LE
- GetPigGrazing()
  : LE
- GetPoison()
  : LE
- GetPoly()
  : LE
- GetQuality()
  : skTTerritory
- GetRain()
  : Weather
- GetRainPeriod()
  : Weather
- GetRodenticide()
  : RodenticideManager
- GetRotIndex()
  : LE
- GetRunNum()
  : LE
- GetSignal()
  : LE
- GetSkScrapes()
  : VegElement
  , LE
- GetSnow()
  : Weather
- GetSoilType()
  : LE
- GetstartDay()
  : BaitLocation
- GetStartValue()
  : CropData
- GetSubType()
  : LE
- GetTemp()
  : Weather
- GetTempPeriod()
  : Weather
- GetTerritoriesByDistance()
  : SkTerritories
- GetTotalN()
  : PositionMap
- GetTotalSpace()
  : PositionMap
- GetTrafficLoad()
  : LE
  , LargeRoad
  , SmallRoad
- gettype()
  : CfgInt
  , CfgBool
- GetType()
  : Farm
- gettype()
  : CfgFloat
  , CfgStr
  , CfgBase
- GetUnsprayedMarginPolyRef()
  : LE
- GetUseFlag()
  : BaitLocation
- GetValidX()
  : LE
- GetValidY()
  : LE
- GetValue()
  : BinaryMapBase
- GetVegAge()
  : LE
- GetVegArea()
  : Landscape
- GetVegBiomass()
  : LE
  , VegElement
- GetVegCover()
  : VegElement
  , LE
- GetVegDensity()
  : LE
  , VegElement
- GetVegHeight()
  : VegElement
  , LE
- GetVegHindrance()
  : Skylark\_Adult
- GetVegPatchy()
  : LE
  , VegElement
- GetVegStore()
  : LE
- GetVegType()
  : VegElement
  , LE
- GetVirtualDiameter()
  : skTTerritory
- GetWeatherHindrance()
  : Skylark\_Adult
- GetWeedBiomass()
  : LE
  , VegElement
- GetWeedPercent()
  : CropData
- GetWind()
  : Weather
- GetWindPeriod()
  : Weather
- GetX()
  : BaitLocation
- GetY()
  : BaitLocation
- GetYear()
  : Calendar
- GetYearNumber()
  : Calendar
- GlobalDate()
  : Calendar
- Glyphosate()
  : Farm
- GoodWeather
  : Skylark\_Male
  , Skylark\_Adult
- GreenElement()
  : GreenElement
- Grid
  : SkTerritories
- GrowthRegulator()
  : Farm


- Generated on Thu Jan 10 2013 13:15:39 for ALMaSS Skylark ODdox by
   1.8.1.1
